# Impact of Particle Size and Sintering Temperature on Calcium Phosphate Gyroid Structure Scaffolds for Bone Tissue Engineering

**DOI:** 10.3390/jfb15120355

**Published:** 2024-11-21

**Authors:** Romina Haydeé Aspera-Werz, Guanqiao Chen, Lea Schilonka, Islam Bouakaz, Catherine Bronne, Elisabeth Cobraiville, Grégory Nolens, Andreas Nussler

**Affiliations:** 1 Siegfried Weller Research Institute, Department of Trauma and Reconstructive Surgery, Eberhard Karls University Tübingen, BG Trauma Center Tübingen, 72076 Tübingen, Germany; rominaaspera@hotmail.com (R.H.A.-W.); guanqiaochen0@gmail.com (G.C.); lea.schilonka@gmx.de (L.S.); 2 CERHUM-PIMW, 4000 Liège, Belgium; bouakaz.abdeldjalil@cerhum.com (I.B.); catherine.bronne@cerhum.com (C.B.); elisabeth.cobraiville@cerhum.com (E.C.); gregory.nolens@cerhum.com (G.N.)

**Keywords:** bone graft, calcium phosphate scaffolds, osteoblast-like cells, osteoclast-like cells, sintering

## Abstract

Due to the chemical composition and structure of the target tissue, autologous bone grafting remains the gold standard for orthopedic applications worldwide. However, ongoing advancements in alternative grafting materials show that 3D-printed synthetic biomaterials offer many advantages. For instance, they provide high availability, have low clinical limitations, and can be designed with a chemical composition and structure comparable to the target tissue. This study aimed to compare the influences of particle size and sintering temperature on the mechanical properties and biocompatibility of calcium phosphate (CaP) gyroid scaffolds. CaP gyroid scaffolds were fabricated by 3D printing using powders with the same chemical composition but different particle sizes and sintering temperatures. The physicochemical characterization of the scaffolds was performed using X-ray diffractometry, scanning electron microscopy, and microtomography analyses. The immortalized human mesenchymal stem cell line SCP-1 (osteoblast-like cells) and osteoclast-like cells (THP-1 cells) were seeded on the scaffolds as mono- or co-cultures. Bone cell attachment, number of live cells, and functionality were assessed at different time points over a period of 21 days. Improvements in mechanical properties were observed for scaffolds fabricated with narrow-particle-size-distribution powder. The physicochemical analysis showed that the microstructure varied with sintering temperature and that narrow particle size distribution resulted in smaller micropores and a smoother surface. Viable osteoblast- and osteoclast-like cells were observed for all scaffolds tested, but scaffolds produced with a smaller particle size distribution showed less attachment of osteoblast-like cells. Interestingly, low attachment of osteoclast-like cells was observed for all scaffolds regardless of surface roughness. Although bone cell adhesion was lower in scaffolds made with powder containing smaller particle sizes, the long-term function of osteoblast-like and osteoclast-like cells was superior in scaffolds with improved mechanical properties.

## 1. Introduction

Bone tissue provides the structural framework for the body and supports other tissues and organs. It protects vital internal organs from damage, such as the skull protecting the brain and the ribcage protecting the heart and lungs [1]. However, bone tissue is not just a static support structure but a dynamic, living tissue that plays vital roles in movement, protection, blood cell production, mineral homeostasis, and various metabolic processes [2].

In contrast to many other organs, bone is able to repair and regenerate itself after trauma thanks to the synchronized function of different cell types, including immune cells, mesenchymal stem cells, chondrocytes, osteoblasts, and osteoclasts [3,4]. However, under conditions and diseases such as cigarette smoking, diabetes, liver damage, and osteoporosis, there is an imbalance in the homeostasis of bone cells, resulting in delayed healing of bone trauma or defects [5,6,7,8].

The increasing aging of the population has a significant impact on bone health, leading to an increased prevalence of bone-related diseases, changes in bone structure and composition, increased risk of fractures and cancer, and increased healthcare needs [9]. This highlights the importance of bone replacement materials, which play a critical role in orthopedic procedures where bone grafting is frequently required to improve or maintain bone health after a fracture.

Although there are several types of bone grafts, autografts are considered the gold standard. They are harvested from the patient’s own body, typically from sites such as the hip, chin, or jaw, and have several advantages such as a high success rate, are well tolerated by the body, contain live osteogenic cells (which contribute to bone formation), and provide osteoconductive and osteoinductive properties. However, they also have several disadvantages, such as limited supply, donor site morbidity and complications (pain, infection risk, etc.), and increased surgical complications [10]. In terms of bone scaffold structure, the gyroid structure has gained importance in recent years due to its high surface-to-volume ratio [11]. Gyroid structures are mathematically defined triple periodic minimal surfaces that can mimic the trabecular architecture of natural bone [12]. This biomorphic design helps to better replicate the structure and properties of the native bone extracellular matrix and can be found in several biological systems such as butterfly wing scales, cell membranes of certain lipid systems, and exoskeletons of some beetle species [13,14].

Hydroxyapatite was chosen for bone scaffold manufacturing over other calcium phosphates, such as tricalcium phosphate, because of its ability to closely resemble the mineral component of natural bone tissue [15,16]. It has a chemical formula of Ca_10_(PO_4_)_6_(OH)_2_ and a Ca/P molar ratio of 1.67, which is almost identical to the inorganic crystalline mineral found in human bone and teeth [17]. This structure and composition allow the scaffolds to integrate with native bone. In addition, hydroxyapatite has excellent biocompatibility and bioactivity in the body [15,18]. It does not trigger immune responses or rejection when implanted. Furthermore, the osteoconductive nature of hydroxyapatite promotes bone cell adhesion, proliferation, and new bone formation at the implant site [16,18].

Based on the previous studies, the particle size of the scaffold powder is an important consideration in bone tissue engineering as it influences the microporosity and mechanical properties of the scaffolds [19,20]. However, the sintering temperature also influences the microporosity and mechanical properties of the scaffold [21]. During the sintering process, materials are heated to temperatures below their melting point, causing atoms to diffuse across particle boundaries and fuse together. Higher sintering temperatures generally result in decreased overall porosity, reduced microporosity in the pore walls, and increased stiffness [22]. This study aimed to optimize hydroxyapatite gyroids previously used in specific clinical cases [23] (powder A detailed in the Materials and Methods section), initially sintered at 1230 °C, by investigating the ability of a smaller grain size to improve mechanical properties without significantly affecting their biological performance.

In bioceramics, it is well-known that finer grains help reduce crack propagation due to the increased number of grain boundaries, which serve as natural barriers to microcrack development [24]. Additionally, a smaller grain size can enhance the reactivity of the powder, thereby potentially improving the biological interactions of the final ceramic, which could enhance the biological performance of calcium phosphate ceramics [25].

To address this, the hydroxyapatite gyroids used for clinical cases (sintered at 1230 °C) were compared with two additional groups of gyroids manufactured from a hydroxyapatite powder with a finer grain size and sintered at 1210 °C and 1250 °C, respectively. The sintering at 1210 °C was chosen to explore the possibility of maintaining mechanical properties while improving biological reactivity by using a lower sintering temperature. Conversely, sintering at 1250 °C was intended to investigate potential improvements in mechanical strength through increased densification, while preserving biological performance due to the finer grain size.

This approach allowed us to assess the impact of grain size on the mechanical and biological properties of hydroxyapatite gyroids, taking into account the specific effects of sintering temperature on densification and surface reactivity, which are crucial factors in the context of bioceramics for clinical applications.

## 2. Materials and Methods

### 2.1. Chemical and Reagents

All chemicals were purchased from Sigma-Aldrich (St. Louis, MO, USA) or Carl Roth (Karlsruhe, Germany). Cell culture medium and supplements were purchased from Sigma-Aldrich or Gibco (Thermo Fisher Scientific, Waltham, MA, USA).

### 2.2. Scaffold Production

**Figure 1:**
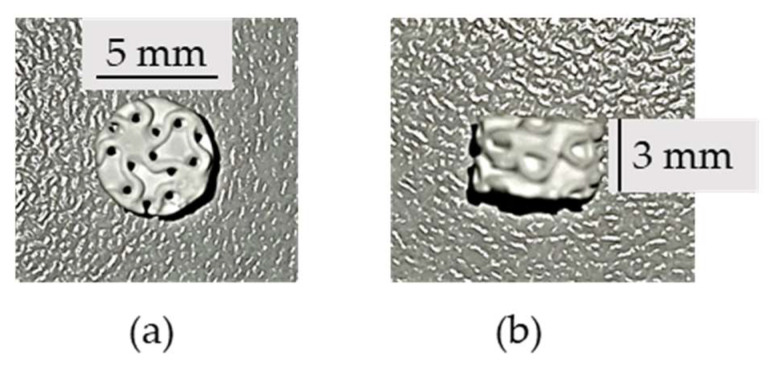
Scaffold with cylindrical shape and gyroid structure. (**a**) Top view. (**b**) Side view.

The scaffolds were designed in the form of gyroids, in accordance with a previous study [26], to achieve a pore size of 900 µm, a wall thickness of 200 µm, and an overall porosity of 66%. Hydroxyapatite scaffolds with a cylindrical shape (height: 3 mm; diameter: 5 mm) and gyroid structure were provided and manufactured by Cerhum using a stereolithography 3D printing machine (V6000 ProMaker, Prodways, Montigny-le-Bretonneux, France) (Figure 1). Scaffolds were fabricated via layer-by-layer 3D printing from a slurry composed of M100T01 resin (CERHUM SA, Liege, Belgium) and hydroxyapatite powder synthesized through microfluidic coprecipitation, following the method outlined by Bouakaz et al. [26]. Scaffolds were then subjected to a debinding process to remove the remaining resin, followed by sintering to promote particle bonding. The sintering temperatures and powders used for each scaffold are summarized in Table 1. Before implementing the scaffold in vitro, they were autoclaved at 134 °C for 18 min [27,28]. As a final step, scaffolds were incubated for 48 h in cell culture medium supplemented with 5% FBS as a sterile control and pre-conditioned before cell seeding [29].

### 2.3. Powder and Scaffold Characterization

#### 2.3.1. Particle Sizes

Particles size analysis was performed using the FRAUNHOFER model for laser beam diffraction analysis according to ISO 13320 (2020) [30] with the laser particle size analyzer BECKMAN COULTER LS 13320-MET-42 (Pasadena, CA, USA).

#### 2.3.2. Chemical Characterization

Powder composition was assessed by X-ray diffraction (XRD) according to ISO 13779-6 [31] with the X-ray diffractometer INEL-EQUINOX 3000 with detector CPS 120—MIC-03 (Thermo Fisher, Waltham, MA, USA).

#### 2.3.3. Mechanical Test

Scaffold compression tests (indentation) were performed according ISO13175 [32] with a velocity of 0.5 mm/min using a Shimadzu AG-X Plus/100kN (Kyoto, Japan).

#### 2.3.4. Scanning Electron Microscopy (SEM)

The scaffold surface microstructure was visualized using a scanning electron microscope (FEI QUANTA 450, Waltham, MA, USA). Following the previous examination, the scaffolds were coated with Scancoat 6 Sputter Coater (Manor Royal, Crawley, West Sussex, UK) for 3 min after attachment to SEM tubs.

### 2.4. Cell Lines

The human monocytic cell lines derived from an acute monocytic leukemia patient, THP-1 cells, were used as osteoclast precursor cells and obtained from the Deutsche Sammlung von Mikroorganismen und Zellkulturen ([DSMZ], Leibniz, Germany). THP-1 cells were cultured with Roswell Park Memorial Institute medium ([RPMI 1640]; Ref. R8758, Merck, Darmstadt, Germany) supplemented with 5% fetal bovine serum ([FBS]; 41G7141K, Thermo Fisher Scientific, Waltham, MA, USA) [33].

The human immortalized mesenchymal stem cells isolated from bone marrow, SCP-1 cells, were used as osteogenic precursor cells (kindly provided by Prof. Dr. Matthias Schieker). SCP-1 cells were cultured with Minimum Essential Eagle’s Medium Alpha (MEM α; 22561-054, Thermo Fisher Scientific, Waltham, MA, USA) supplemented with 5% FBS, negative tested (by RT-PCR) for CD14 and CD34, and positive tested for CD73, CD90, and CD105 [33].

Both cell types were cultured at 37 °C with 100% humidity and a 5% CO_2_ incubator. The cell culture medium was replaced twice a week, and cells were subcultured with 80–90% confluency.

### 2.5. Bone Cell Seeding and Culture

#### 2.5.1. Osteoblast-like Cell 3D Monoculture

An SCP-1 cell monoculture was formed by seeding 1 × 10^4^ cells/12 µL per scaffold in MEMα culture medium. Cells were incubated at 37 °C for 4 h to allow attachment before adding 500 µL MEMα culture medium. The following day, the medium was replaced to induce osteogenic differentiation by MEM α, supplemented with 2% FBS, 200 µM L-ascorbic acid 2-phosphate (A8960-5G, Sigma), 5 mM β-glycerolphosphate (G9422-10, Sigma, St. Louis, MO, USA), 25 mM HEPES (HN78.2, Carl Roth, Karlsruhe, Germany), 1.5 mM CaCl_2_ (CN93.1, Carl Roth, Karlsruhe, Germany), and 5 µM cholecalciferol (95230, Sigma, St. Louis, MO, USA). During the osteogenic differentiation process, the medium was replaced every 3–4 days and scaffolds were collected after culturing for 24 h, 48 h, 7 days, 14 days, and 21 days for further analysis (attachment, number of live cells, and osteoblast-like cell function).

#### 2.5.2. Osteoclast-like Cell 3D Monoculture

THP-1 cell monoculturing was carried out by seeding 8 × 10^4^ cells/12 µL per scaffold in RPMI culture medium. To form a macrophage-like phenotype of THP-1 cells, 200 nM phorbol 12-myristate 13-acetate (PMA; Ref. AB120297, Abcam, Cambridge, UK) was also added [29]. Cells were incubated at 37 °C for 4 h to allow attachment before adding 500 µL RPMI culture medium. After 24 h culturing, scaffolds were collected for further analysis (attachment and number of live cells).

#### 2.5.3. Osteoblast- and Osteoclast-like Cell 3D Co-Culture

A bone co-culture system was formed by seeding THP-1 8 × 10^4^ cells/12 µL per scaffold in a 50:50 mix of MEMα:RPMI supplemented with 2% FBS, 200 µM L-ascorbic acid 2-phosphate, 5 mM β-glycerolphosphate, 25 mM HEPES, 1.5 mM CaCl2, 5 µM cholecalciferol (bone differentiation medium), and 200 nM PMA. Cells were incubated at 37 °C for 4 h to allow attachment before adding 500 µL of bone differentiation medium with 200 nM of PMA. The following day, after removing the medium, SCP-1 1 × 10^4^ cells/12 µL per scaffold were seeded in bone differentiation medium (without PMA). After 4 h incubation at 37 °C to allow cell attachment, 500 µL of bone differentiation medium was added. During the cultivation, the medium was replaced every 3–4 days and scaffolds were collected after culturing for 7 days, 14 days, and 21 days for further analysis of the attachment, viability, and osteoblast- and osteoclast-like cell function [29].

### 2.6. Viability and Functional Assays

#### 2.6.1. Mitochondrial Activity—Resazurin Conversion Assay

To assess bone-forming (osteoblast-like) and -resorbing (osteoclast-like) cell viability and proliferation, mitochondrial activity was determined based on the resazurin conversion. Scaffolds were incubated for 2 h at 37 °C with 500 µL of resazurin working solution (0.0025% *w*/*v* in PBS) (Ref. 199,303-1G; Merck). Formed resorufin was quantified fluorometrically with an Omega Plate Reader (ex/em  =  540/590  nm; BMG Labtech, Ortenberg, Germany). Data were corrected to a background control (working solution on scaffolds without cells) [29].

#### 2.6.2. Total DNA

Attachment and proliferation of osteoblast- and osteoclast-like cells were determined by total DNA content. Scaffolds were incubated for 5 min with 250 µL NaOH (50 mM–98 °C; Ref. T135.1, Carl Roth) and frozen overnight at −20 °C. Then, scaffolds were incubated for 15 min at 60 °C and pH was neutralized with the addition of 1 M TRIS (pH = 8, Ref. N C4706, Sigma). The solution was transferred to a V-bottomed 96-well plate, and impurities were removed by centrifugation at 2000× *g*-10 min. Total DNA was quantified photometrically using an LVIS plate (BMG Labtech, Ortenberg, Germany) and Omega Plate Reader (wavelength λ = 230 nm, λ = 260 nm, and λ = 280 nm; 25 flashes [BMG Labtech, Ortenberg, Germany]) [29].

#### 2.6.3. Calcein AM Staining

Viable osteoblast- and osteoclast-like cells were visualized by intracellular esterase activity using calcein-AM staining. Scaffolds were incubated with calcein-AM (2 µmol/L; Ref. N 17783, Sigma) and Hoechst 33342 (1:1000 in PBS; Ref. N B2261) at 37 °C for 30 min. Microscopy images were acquired (epifluorescence: EVOS FL, life technologies, Darmstadt, Germany) after removing remained dye with PBS [33].

#### 2.6.4. Osteoblast-like Cell Function Alkaline Phosphatase (AP) Activity

Osteoblast-like cell (bone-forming) function was determined by alkaline phosphatase (AP) activity. Scaffolds were incubated for 2 h at 37 °C with 500 µL of working solution (0.2% *w*/*v* 4-nitrophenyl-phosphate (Ref. N7660, Merck), 50 mM glycine (Ref. 3908.2, Carl Roth), 1 mM MgCl_2_ (Ref. 3908.2, Carl Roth), and 100 mM TRIS (pH 10.5)). Formed 4-nitrophenol was quantified photometrically with an Omega Plate Reader (λ = 405 nm; BMG Labtech). Data were corrected to a background control (working solution on scaffolds without cells) and normalized to the cell number by the total DNA (isolated from different scaffolds) [29].

#### 2.6.5. Osteoclast-like Cell Function Carbonic Anhydrase II (CAII) Activity

Osteoclast-like cell (bone-resorbing) function was determined by carbonic anhydrase II (CAII) activity. Scaffolds were incubated for 15 min at 37 °C with 500 µL of working solution (10 mM TRIS, 60 mM sodium chloride [Ref. S7653, Carl Roth], and 200 mM 4-nitrophenyl acetate). Formed 4-nitrophenol was quantified photometrically with an Omega Plate Reader (λ = 405 nm; BMG Labtech). Data were corrected to a background control (working solution on scaffolds without cells) and normalized to the cell number by the total DNA (isolated from different scaffolds) [29].

#### 2.6.6. Osteoclast-like Cell Function Tartrate-Resistant Acid Phosphatase (TRAP) Activity

Osteoclast-like cell (bone-resorbing) function was determined by tartrate-resistant acid phosphatase (TRAP) activity. Scaffold supernatants (30 µL) were incubated for 6 h at 37 °C with 90 µL working solution (0.2% *w*/*v* 4-nitrophenyl phosphate, 100 mM sodium acetate [Ref. X891.2, Carl Roth], and 50 mM sodium tartrate [Ref. 4165.1, Carl Roth], pH 5.5). Formed 4-nitrophenol was quantified photometrically with an Omega Plate Reader (λ = 405 nm; BMG Labtech) after adding 90 μL of 1 M NaOH (Ref. T135.1, Carl Roth). Data were corrected to a background control (working solution, NaOH, and culture medium) on scaffolds without cells) and normalized to the cell number by the total DNA [29].

#### 2.6.7. Dot Plot

Matrix formation and resorption were determined by protein levels of procollagen type I N-terminal propeptide (PINP) and collagen type I N-telopeptide (NTX) levels in the culture supernatant. Scaffold supernatant (60 µL) was transferred to a nitrocellulose membrane using a 96-well dot blotter (Ref. T790.1 Carl Roth, Karlsruhe, Germany). After blocking the membrane for 1 h with 5% albumin (Ref. 8076.4, Carl Roth) and washed with TBS-T, the membrane was incubated overnight at 4 °C with primary antibodies (Table 2) in TRIS-buffered saline/Tween 20 (TBS-T) containing 10 mM TRIS (pH 7.6), 0.15 mM NaCl (Ref. N 27810.295, AnalaR NORMAPUR), and 0.1% *v*/*v* Tween-20 (Ref. 9127.1, Carl Roth). Following washing with TBS-T, the membrane was incubated for 2 h with secondary antibody. All antibodies were diluted in TBS-T and are summarized in Table 2. For signal development, membranes were incubated for 1 min with an electrogenerated chemiluminescence (ECL) substrate solution (100 nM TRIS, 250 mM Luminol [Ref. 4203.1, Carl Roth], 90 mM p-coumaric acid [Ref. 9908.1, Carl Roth], 30% *v*/*v* H2O2 [Ref. CP26.5, Carl Roth]). ImageJ software Version 1.54 was used to quantify the detected chemiluminescent signals by a charge-coupled device camera (INTAS Science Imaging, Göttingen, Germany) [33].

#### 2.6.8. Statistical Analysis

The statistical analysis and data plots were achieved using the GraphPad Prism software (GraphPad Software 9.0, La Jolla, CA, USA). Datasets were represented in a scatter dot plot displaying all data points, the mean, and the standard deviation of the mean, or a symbol or bar plot displaying the mean and standard deviation of the mean. Results acquired through in vitro experiments were obtained with three biological replicates (*n* = 3 independent times) and a minimum of two technical replicates (*n* ≥ 2, for each type of scaffold tested). Datasets were compared using a two-way analysis of variance test followed by Tukey’s multiple comparisons, or the Kruskal–Wallis H test (non-parametric multiple groups) followed by Dunn’s multiple-comparison test. A *p*-value < 0.05 was considered statistically significant. The statistical tests are indicated in each figure legend.

## 3. Results

### 3.1. Powder B Showed a Narrow Particle Size Distribution Compared to Powder A

In Table 3, we present the particle size distributions of powder A and powder B. The powders show no difference in the smallest particle size, with 10% of the particles being smaller than 1.7 µm. However, the median particle size (D50) was smaller in powder B than in powder A. Furthermore, 90% of the particles were approx. 2-fold smaller in powder B compared with those in powder A.

### 3.2. Powder B Is Not Different in Composition from Powder A

**Figure 2:**
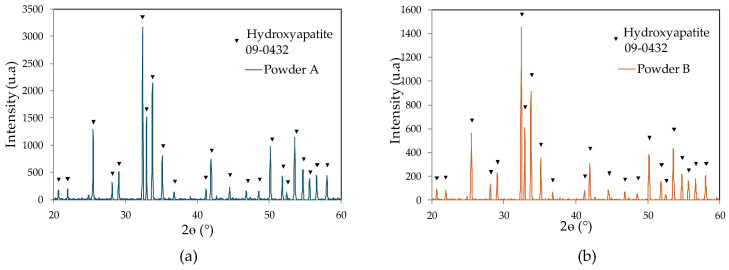
Powder composition determined by X-ray diffractometry (XRD). Representative XRD curve for (**a**) powder A and (**b**) powder B.

The X-ray diffractometry analysis results are summarized in Table 4 and Figure 2. The results show no difference in organic residues between the powders. The detected levels of total organic carbon and hydrocarbon were less than 1 mg/L for both powders. These results show that there is no residual resin present that could interfere with the determination of the particle sizes of the powders. Additionally, only approx. 1% tricalcium phosphate α was detected in the compositions of both powders. Tricalcium phosphate β, tetracalcium phosphate, and calcium oxide were not detected in their compositions. However, powder B had a lower level of inorganic residues and a larger crystallinity ratio than powder A.

### 3.3. Powder B Improves Scaffold Mechanical Strength Compared to Powder A

**Figure 3:**
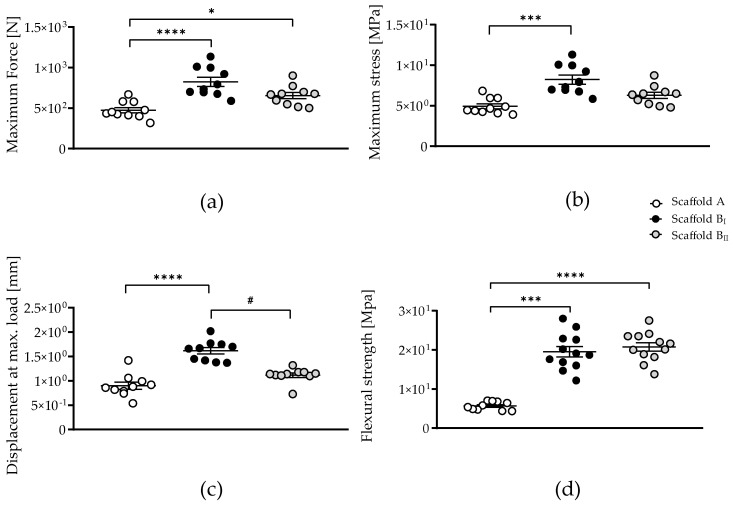
Mechanical characterization of the three scaffolds tested. (**a**) Maximum force, (**b**) maximum stress, (**c**) displacements at maximum load, and (**d**) flexural strength were analyzed on scaffolds generated with powder A sintering at 1230 °C [Scaffold A] or powder B sintering at 1250 °C and 1210 °C [scaffolds B_I_ and B_II_, respectively]. The data are presented as the mean, standard error of the mean, and all data points. Data were analyzed by the Kruskal–Wallis test followed by Dunn’s multiple comparisons. *p*-values are classified as * *p* < 0.05; *** *p* < 0.001; and **** *p* < 0.0001 for comparison between scaffold B and scaffold A and as # *p* < 0.05 for comparison within scaffold B.

The results of the mechanical tests carried out on scaffold B compared to scaffold A are shown in Figure 3. The maximum force was significantly lower for scaffold A compared to those for scaffolds B_I_ (*p* < 0.0001) and B_II_ (*p* = 0.0412) (Figure 3a). However, only scaffold B_I_ (sintered at a high temperature) showed a significant increase in maximum stress (*p* = 0.0001) and displacement at the maximum load (*p* < 0.0001) compared to those for scaffold A (Figure 3b,c). Scaffold B_II_ (sintered at a low temperature) had a significantly lower displacement at the maximum load (*p* = 0.0100) compared to that for scaffold B_I_ (sintered at a high temperature). The flexural strength was significantly lower in scaffold A; scaffold B demonstrated increased flexural strength regardless of the sintering temperature (B_I_ *p* = 0.0006 and B_II_ *p* < 0.0001). The results demonstrate that scaffolds generated with powder B provide a significant improvement in mechanical strength and flexural strength values compared to those generated with powder A.

### 3.4. Powder B Leads to Scaffolds with Smaller Micropores and a Smoother Surface Compared to Those Formed with Powder A

**Figure 4:**
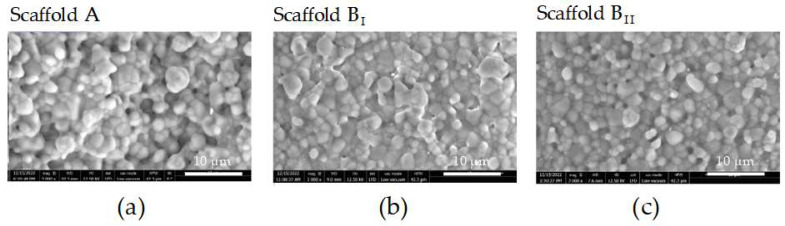
Surface topographies of the three scaffolds analyzed by scanning electron microscopy (SEM). (**a**) Scaffold generated with powder A and sintering at 1230 °C. (**b**) Scaffold generated with powder B and sintering at 1250 °C. (**c**) Scaffold generated with powder B and sintering at 1210 °C (scale bar 10 µm).

SEM images exhibited an irregular microtopography with several micropores and heterogeneous surfaces for all scaffolds (Figure 4). Scaffold A had bigger micropores and a rougher surface compared to scaffolds prepared with powder B (Figure 4a). Scaffold B_II_ had more micropores than scaffold B_I_ (Figure 4b,c).

### 3.5. Powder B Scaffolds Lead to Lower Osteoblast Precursor Cell Attachment Compared to Powder A Scaffold

**Figure 5:**
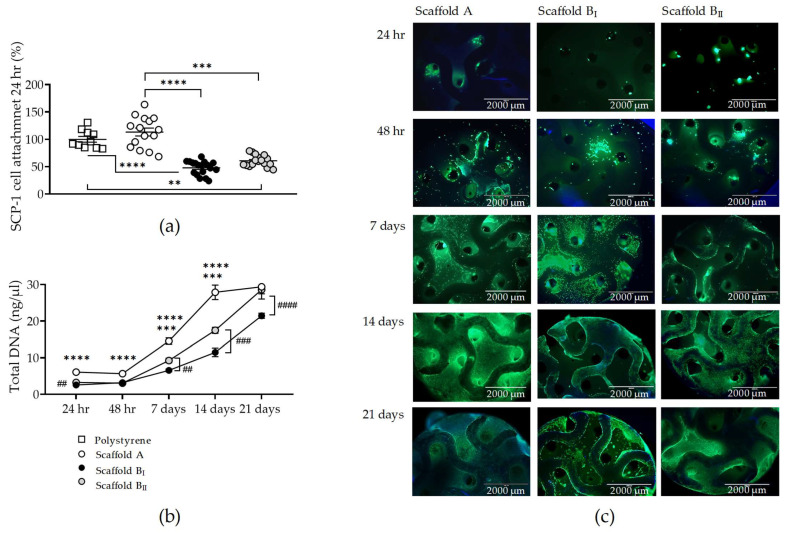
SCP-1 cell attachment, number of live cells, and proliferation on three scaffolds tested. SCP-1 cells were seeded and cultured on scaffolds A, B_I_, and B_II_ for 21 days. (**a**) Attached SCP-1 cells on scaffolds compared to cultured polystyrene. Number of live SCP-1 cells were analyzed after 24 h, 48 h, 7 days, 14 days, and 21 days by total DNA levels (**b**) and visualized by esterase activity (**c**) using calcein-AM (green) and nuclear staining using Hoechst 33342 (blue) (scale bar 2000 µm). Each measure was conducted at least three independent times in triplicate. The data are presented as the mean, standard error of the mean, and all data points. Data were analyzed by the Kruskal–Wallis test followed by Dunn’s multiple comparisons (**a**) or a two-way analysis of variance test followed by Tukey’s multiple comparisons (**b**). *p*-values are classified as ** *p* < 0.01; *** *p* < 0.001; and **** *p* < 0.0001 for comparison between scaffold B and scaffold A and as ## *p* < 0.01; ### *p* < 0.001; and #### *p* < 0.0001 for comparison within scaffold B.

In order to evaluate and compare the biocompatibility of scaffolds generated with powder B (narrow particle size distribution) and powder A, osteoblast-like SCP-1 cells (osteoblast precursor) were cultured under osteogenic conditions on the three scaffolds. After 24 h, the SCP-1 cell attachment rate to scaffold A was similar to cultured polystyrene. However, the adherence of SCP-1 cells was significant lower for scaffolds B_I_ (*p* < 0.0001) and B_II_ (*p* = 0.0001) compared to that for scaffold A. No differences were detected in SCP-1 cell attachment between scaffolds B_I_ and B_II_ (Figure 5a). Total DNA levels and live staining confirmed the previous attachment results, with significantly less DNA isolated from cells cultured on scaffold B compared to those cultured on scaffold A up to day 14 (Figure 5b,c). Interestingly, no cytotoxicity was detected by any of the scaffolds evaluated, and SCP-1 cell proliferation (increasing DNA levels and esterase activity with culture time) was found in all scaffolds tested (Figure 5b,c). No significant differences in SCP-1 cell number were detected after a 21-day culture on scaffolds A and B_II_ (Figure 5b,c).

### 3.6. Scaffold Powder B Enhances the Osteogenic Potential of the Osteoprogenitor Compared to Powder A

**Figure 6:**
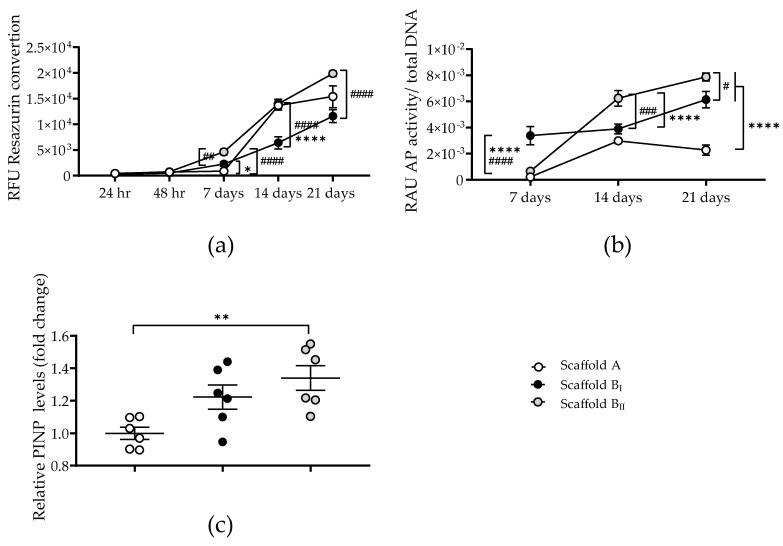
SCP-1 osteogenic differentiation potential on three scaffolds tested. SCP-1 cells were seeded and cultured under osteogenic condition on scaffolds A, BI, and BII for 21 days. (**a**) Metabolic activity of SCP-1 cells were analyzed after 24 h, 48 h, 7 days, 14 days, and 21 days by mitochondrial activity as relative fluorescence units (RFU). (**b**) Alkaline phosphatase (AP) activity normalized to DNA of SCP-1 cells were analyzed after 7 days, 14 days, and 21 days as relative absorbance units (RAU). (**c**) Procollagen type I N-propeptide (PINP) supernatant levels were determined after 21-day osteogenic culture. Each measure was conducted at least three independent times in duplicate. The data are presented as the mean, standard error of the mean, and all data points. Data were analyzed by a two-way analysis of variance test followed by Tukey’s multiple comparisons. *p*-values are classified as * *p* < 0.05; ** *p* < 0.01; and **** *p* < 0.0001 for comparison between scaffold B and scaffold A and as # *p* < 0.05; ## *p* < 0.01; ### *p* < 0.001; and #### *p* < 0.0001 for comparison within scaffold B.

To assess the osteoinductivity of scaffold powder B compared to powder A, SCP-1 cell osteogenic potential was analyzed by the early osteogenic differentiation marker AP and the bone formation marker PINP. Mitochondrial activity demonstrated that SCP-1 cells under osteogenic differentiation are metabolically active for 21 days in the three scaffolds. After 21 days, the osteogenic culture mitochondrial activity and total DNA from SCP-1 cells showed no significant differences between scaffold A and scaffold B. Interestingly, SCP-1 cells cultured on scaffold B_I_ (sintered at a high temperature) showed a significantly lower mitochondrial activity and total DNA compared to those cultured on scaffold B_II_ (Figure 5b and Figure 6a).

The AP activity of the SCP-1 cell culture on scaffold B was significant higher compared to that on scaffold A. Although on day 7, SCP-1 cells cultured on scaffold B were reduced compared to those on scaffold A, the metabolic and AP activity detected was higher in scaffold B than in scaffold A (Figure 6b). This increased osteoblast activity in scaffold B was also reflected in the levels of PINP secreted by the osteoblast-like cells. Between scaffolds B_I_ and B_II_ (same particle size), there were no significant differences in PINP secretion levels; however, scaffold B_II_ (sintered at a low temperature) showed a significant increase in osteoblast-like cell activity on days 14 and 21 compared to scaffold B_I_.

### 3.7. All Scaffolds Showed Lower Osteoclast Precursor Cell Attachment than That for Polystyrene

**Figure 7:**
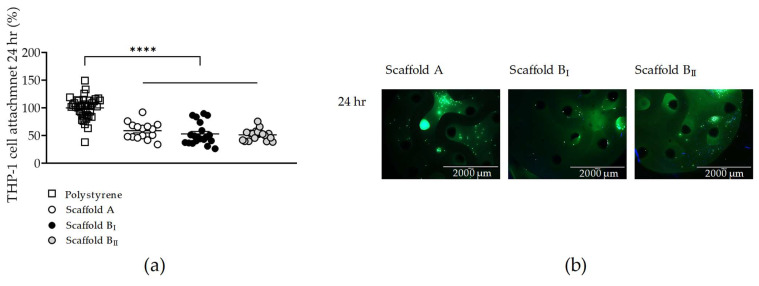
THP-1 cell attachment and number of live cells on three scaffolds tested. THP-1 cells were seeded and cultured on scaffolds A, BI, and BII for 24 h. (**a**) Attached SCP-1 cells on scaffolds compared to cultured polystyrene. Number of live THP-1 cells were visualized after 24 h by esterase activity (**b**) using calcein-AM (green) and nuclear staining using Hoechst 33342 (blue) (scale bar 2000 µm). Each measure was conducted at least three independent times in triplicate. The data are presented as the mean, standard error of the mean, and all data points. Data were analyzed by the Kruskal–Wallis test followed by Dunn’s multiple comparisons. *p*-values are classified as **** *p* < 0.0001 for comparison between scaffold B and scaffold A.

Since scaffolds generated with powder B (narrow particle size distribution) and A showed no cytotoxic effects on osteoblast-like SCP-1 cells, we tested the biocompatibility of the scaffolds on osteoclast-like THP-1 cells (osteoclast precursor). After 24 h, the attachment rate of THP-1 cells to all tested scaffolds was 50%, significantly lower than culture polystyrene. However, no significant differences in THP-1 cell adherence were found between the different scaffolds tested (Figure 7a). Live THP-1 cells were visualized in all the scaffolds at 24 h after cell seeding (Figure 7b). The results showed that no scaffolds evaluated, regardless of particle size distribution or sintering temperature, had cytotoxic effects on osteoclast precursor cells.

### 3.8. Powder B Scaffolds Maintain the Viability of Osteoclasts and Osteoblasts in a Direct Bone Co-Culture System

**Figure 8:**
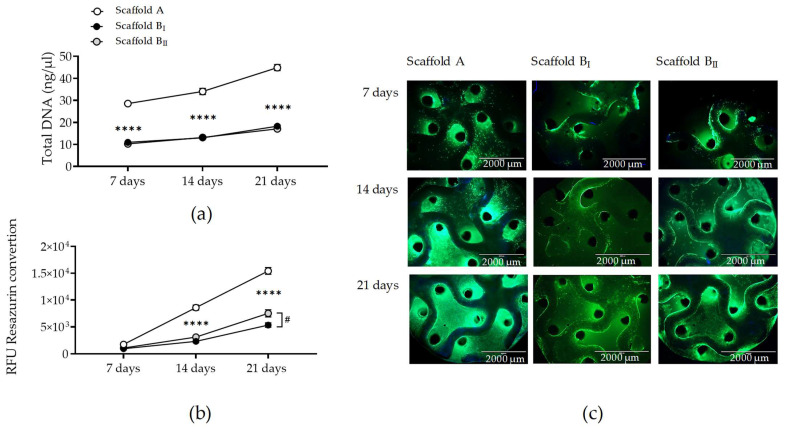
Bone cell viability and proliferation in co-cultures on the three scaffolds tested. THP-1 and SCP-1 were seeded and co-cultured on scaffolds A, BI, and BII for 21 days. Quantification of total DNA (**a**) and mitochondrial activity by resazurin conversion (**b**) in bone co-cultures after 7 days, 14 days, and 21 days. Number of live bone co-cultures were visualized by esterase activity (**c**) using calcein-AM (green) and nuclear staining with Hoechst 33342 (blue) (scale bar 2000 µm). Each measure was conducted at least three independent times in triplicate. The data are presented as the mean, standard error of the mean, and all data points. Data were analyzed by two-way analysis of variance test followed by Tukey’s multiple comparisons. *p*-values are classified as **** *p* < 0.0001 for comparison between scaffold B and scaffold A and as # *p* < 0.05 for comparison within scaffold B.

Since scaffolds generated with powder B were biocompatible with osteoblast and osteoclast precursor cells in a monoculture, we were interested in evaluating the viability of bone cells cultured in a co-culture on the scaffold powders A and B for 21 days. Osteoclast-like THP-1 and osteoblast-like SCP-1 cells were directly co-cultured on the three scaffolds. Total DNA levels were lower for co-cultures on scaffold powder B than those on scaffold powder A at all time points (*p* < 0.0001). However, DNA levels correlated positively with the culture time for the co-cultures in scaffold powders A and B, demonstrating bone cell proliferation in all scaffolds tested (Figure 8a). While the mitochondrial activity in co-cultures increased in a time-dependent manner for all scaffolds, that in scaffold powder B was significantly lower on days 14 and 21 compared to that in scaffold powder A. Significant higher metabolic activity (*p* = 0.0235) was detected in scaffold powder B_II_ (sintered at a lower temperature) co-cultures compared to scaffold powder B_I_ co-cultures on day 21 (Figure 8b). The previous results were supported by these co-cultures, as determined by esterase activity staining (Figure 8c).

### 3.9. Powder B Scaffolds Increase Bone Resorption Activity in a Direct Bone Co-Culture System Compared to Powder A Scaffolds

**Figure 9:**
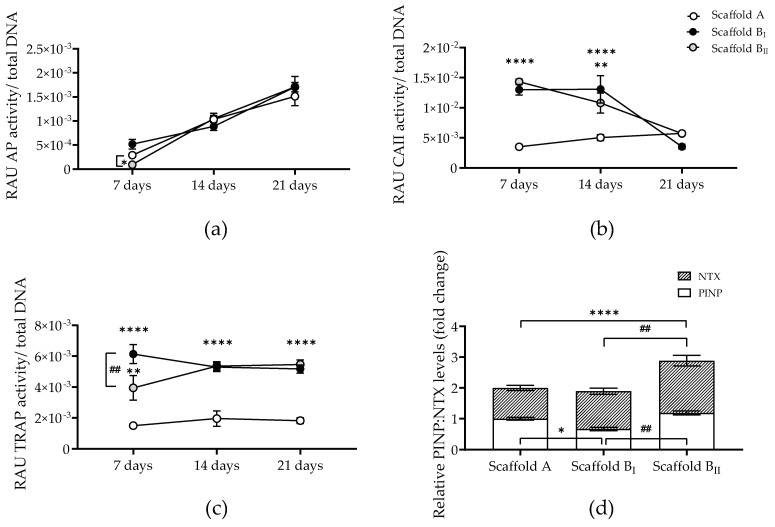
Osteoblast- and osteoclast-like cell function in co-cultures on the three scaffolds tested. THP-1 and SCP-1 were seeded and co-cultured on scaffolds A, BI, and BII for 21 days. (**a**) Alkaline phosphatase (AP), (**b**) carbonic anhydrase II (CAII), and (**c**) tartrate-resistant acid phosphatase (TRAP) activity normalized to DNA of bone co-cultures were analyzed after 7 days, 14 days, and 21 days as relative absorbance units (RAU). (**d**) Procollagen type I N-propeptide (PINP) and collagen type I N-telopeptide (NTX) supernatant levels were determined after a 21-day culture. Each measure was conducted at least three independent times in duplicate. The data are presented as the mean or standard error of the mean. Data were analyzed by a two-way analysis of variance test followed by Tukey’s multiple comparisons. *p*-values are classified as **** *p* < 0.0001, ** *p* < 0.01, and * *p* < 0.05 for comparison between scaffold B and scaffold A and as ## *p* < 0.01 for comparison within scaffold B.

In order to evaluate and compare the osteoconductivity of scaffold powder B and powder A, the osteoblast-like cell activity of the co-cultures was determined by the AP, CAII, and TRAP activity on days 7, 14, and 21. Additionally, de novo bone formation and resorption were analyzed indirectly by the secretion levels of PINP (type 1 collagen propeptide-formation marker) and NTX (type I collagen N-terminal telopeptide-degradation marker) (Figure 9). Osteoblast-like cell activity (AP) gradually increased with culture time in co-culture scaffolds regardless of the powder (Figure 9a). However, a significant increase in osteoclast activity (CAII and TRAP) was observed in bone co-cultures on powder B scaffolds compared those on powder A scaffolds (Figure 9b,c). Interestingly, CAII activity was significantly higher on days 7 (*p* < 0.0001) and 14 on scaffold powder B compared to on scaffold powder A (*p* B_I_ < 0.0001, *p* B_II_ = 0.0015); nevertheless, on day 21, CAII levels decreased for scaffold powder B to the level of powder A. However, no differences were detected in CAII activity between scaffolds B_I_ and B_II_ (Figure 9b). In addition, TRAP activity was upregulated in co-cultures on scaffold powder B compared to those on powder A for all time points analyzed. Scaffold powder B_I_ (sintered at a high temperature) showed a significantly higher TRAP activity on day 7 compared to scaffold powder B_II_ (*p* < 0.0015). Nevertheless, TRAP activity in scaffold powder B_II_ increased to the level of powder B_I_ on days 14 and 21 (Figure 9c). In addition, NTX levels of bone co-cultures on scaffold powder B were increased compared to those on scaffold powder A (approx. 2-fold for scaffold powder B_I_ and 3-fold for scaffold powder B_II_). This upregulation of NTX levels was significant in bone co-cultures on scaffold powder B_II_ compared to those on scaffold powder A (*p* < 0.0001) and powder B_I_ (*p* = 0.0052) (Figure 9d). The lower secreted PINP levels were measured in the supernatant of bone co-cultures on scaffold B_I_ (*p* = 0.0021 compared to those on scaffold powder B_II_ and *p* = 0.0497 compared to those on scaffold powder A). Similar PINP levels were detected in bone co-cultures on scaffold powder B_II_ compared to those on scaffold powder A (Figure 9d).

## 4. Discussion

Autologous bone remains the gold standard for the correction of bone defects and the treatment of non-union fractures, but research has been underway to investigate several alternatives for bone reconstruction [10]. Many products used to correct traumatic bone defects are available in particulate form, which often lack the structural integrity to provide adequate support and anchoring for bone and immune cells. This has driven a growing interest in developing scaffolds that can offer both support and anchorage. In addition to the material used, the scaffold structure is essential to ensure good cell attachment and differentiation, promoting bone formation, integration with the host tissue, and overall success in treating bone defects.

In the present study, the role of the particle size as well as the sintering temperature on the mechanical properties and biocompatibility of CaP 3D-printed scaffolds was investigated.

Gyroid structures were selected as the scaffold design due to their triple periodic minimal surfaces, which improve fluid access and facilitate cell colonization, nutrient exchange, and waste removal [34,35]. The high surface-to-volume ratio of these structures increases the area available for bone cell adherence and proliferation [36]. In addition, the zero-mean curvature of the gyroid structures enhances the mechanical properties, favoring osteogenic differentiation and making these scaffolds a suitable choice for bone repair [37,38,39].

The successful colonization, proliferation, and differentiation of progenitor bone cells also depend on the pore size of the scaffold. The scaffolds used in this study have a macropore size of 900 µm, as several in silico, in vitro, and in vivo studies have shown that large pores promote bone formation [27,40], while pore sizes smaller than 100 µm favor the formation of unmineralized osteoid [41,42].

In addition to the macropore size of the scaffolds, the microporosity of the material also plays a critical role in affecting its scaffold surface properties, influencing cell colonization, proliferation, and differentiation. Electron microscopy images show micropores on the surface of all scaffolds. Microporosity increases surface area and roughness, providing more sites for protein adsorption or bone cell–scaffold interaction, and also increases the release of degradation ions or biofactors from the scaffold, which could improve bone formation [43,44]. As expected, scaffolds produced with powder B presented smaller micropores due to a narrower particle distribution (smaller grant size) compared to those produced with powder A (larger grant size, spacious particle distribution). This has also previously been demonstrated with other powders [45].

Despite using different powders, the scaffolds in this study maintained a consistent design and macropore size. While both powders share the same inorganic composition, powder A has larger particle sizes than powder B. Particle size plays an important role in affecting scaffold mechanical properties and topography, since a smaller grain size is associated with a narrow distribution of the particles. Mechanical analysis revealed that scaffolds made with powder B, which contains smaller particle sizes, show a significant increase in the modulus of rupture, as was also observed for silica [46] and boron carbide [47] or SiC ceramics [48].

When developing bone scaffolds, the mechanical, osteoinductive, and osteoconductive properties must be considered, including the potential immune response modulated by the size and shape of hydroxyapatite particles. Lebre et al. showed that needle-shaped and smaller hydroxyapatite particles enhanced the innate immune response [49].

However, in addition to the powder particle size, the sintering temperature plays an important role in defining the microporosity and mechanical strength of the scaffold. During the sintering process, which involves heating the scaffold material to a temperature below its melting point, the microstructure and physical properties of the resulting scaffold are significantly affected. Scaffold B_II_, produced at the highest sintering temperature, (1250 °C) was shown to have improved mechanical properties compared to scaffolds A and B_I_, sintered at lower temperatures (1230 °C and 1210 °C, respectively). A higher sintering temperature increases particle bonding and densification, which causes an increase in hardness and elastic modulus [22]. Since scaffold A has a higher sintering temperature than scaffold B_II_, it was expected to have improved mechanical properties. However, our results demonstrated worse mechanical properties for scaffold A, due to the larger particle size compared to scaffold B_II_, which implies that the contact surface between the particles is smaller, causing a weaker bond. Therefore, the design and fabrication of scaffolds for bone tissue engineering play an important role in enhancing their biocompatibility and osteogenic potential through different mechanics.

As our results show, the sintering temperature affects not only the mechanical properties but also the microporosity of the material. Scaffold B_II_ (sintered at 1210 °C) showed a more interconnected pore network compared to scaffold B_I_ (sintered at 1250 °C). This is in line with previous studies on hydroxyapatite [50] and copper porous materials [51], which demonstrated that as the sintering temperature increases, microporosity decreases. As a result of the densification process, particles fuse, eliminating smaller pores first and leaving larger pores.

In addition to having suitable mechanical properties, such as stiffness and load-bearing capacity, the scaffold should be biocompatible and non-toxic, to facilitate in vivo integration after implantation. Biocompatibility analysis demonstrated that no scaffolds showed cytotoxicity to osteoblast-like and osteoclast-like cells, regardless of the powder and sintering temperature. The lowest sintering temperature used in this study was 1210 °C, since a previous study conducted with similar scaffolds showed that at temperatures below 1200 °C, non-cohesive hydroxyapatite particles are detected [52], which may exhibit cytotoxicity due to their high ion reactivity [50].

Regarding the attachment of osteoblast progenitor cells, greater adhesion was observed in scaffold A due to its greater microporosity. Increased microporosity generates stronger capillary forces, which attract cells into the pores, promoting a more homogeneous distribution [53]. It should be noted that to successfully attract cells, the capillary pressure should exceed cells’ critical pressure [54]. Since cells’ critical pressure is closely related to the cell radius and cortical tension, this could explain the lower attachment observed in osteoclast precursor cells compared to osteoblast precursor cells on scaffold A. It should be emphasized that the capillary pressure required to draw different types of cells into the micropores of the scaffolds can be influenced and optimized by the choice of powder particle size and sintering temperature [55]. Interestingly, our results showed an increase in osteoconductivity (increased levels of AP activity and secreted PINP) in scaffolds made with powder B compared to those made with powder A, even though the initial cell attachment was lower in scaffold B.

The choice of cell culture platform significantly impacts osteoclast behavior and longevity. It is reported that a tissue culture on plastic supports reproducible osteoclastogenesis, and that mineralized substrates like hydroxyapatite-coated surfaces or decellularized bone powder may better mimic the in vivo environment and support longer-term cultures [56,57,58,59]. Interestingly, the attachment of osteoclast progenitor cells was lower for all scaffolds included in this study compared to polystyrene. However, after long-term osteoclast co-culturing with osteoblasts, viable and functional osteoclasts were detected after 21 days on scaffold powder B (regardless of their sintering temperature). These results support the notion that scaffold powder B can maintain an osteoclast in vitro culture in the long term, comparable to the in vivo lifespan (between 14 and 21 days [60,61]). This is because the scaffolds used in this study can promote the growth of co-cultured osteoblasts and osteoclasts in bone-like arrangements (observed by the ability to remodel the matrix [detection of collagen formation (PINP) and degradation precursors (NTX)]), as also reported for tailored amorphous multi-porous scaffolds [62]. In this study, only the attachment of osteoclasts was evaluated in the monoculture, while all functional assays were performed in co-cultures with osteoblasts, as optimized in previous studies [29,33]. We wanted to avoid the incorporation of osteoclastogenesis-inducing factors into the system (e.g., receptor activator of nuclear factor-κB ligand or macrophage colony-stimulating factor), ensuring the osteoblasts coordinated osteoclast fusion and differentiation in response to the culture substrate (scaffolds).

These results suggest that the initial cell attachment of bone cells is negatively correlated with their level of osteogenic differentiation, as scaffolds made with powder B showed lower cell adhesion but higher osteoblast- and osteoclast-like cell activity. The lower initial cell attachment may be related to the smoother surface of powder B scaffolds, along with a lower capacity to absorb proteins associated with cell–substrate interaction present in the cell culture medium (e.g., immunoglobulins, fibronectin, etc.). The increase in osteoconductive properties observed with scaffold powder B, despite the smoother surface, may be explained by the fact that surface roughness interacts with other properties, such as surface chemistry and stiffness, to influence cell behavior [63].

It should be noted that coatings with growth factors or proteins that facilitate osteogenic adhesion and differentiation (e.g., bone morphogenic proteins) were not used in this study. Whether such treatments have a beneficial effect on the biocompatibility of bone cells requires further study.

## 5. Conclusions

This study highlights the importance of the sintering temperature and the grain size in the development of hydroxyapatite gyroids for clinical applications. The findings suggest that lower sintering temperatures may enhance the biological performance without compromising the mechanical integrity, while higher temperatures can improve densification and strength. Ultimately, this research underscores the critical balance between mechanical and biological properties in the design of bioceramics, paving the way for optimized materials that can better serve our clinical needs.

## Figures and Tables

**Table 1:** Scaffold sintering temperatures.

Scaffold	Powder	Sintering Temperature
Scaffold A	A	1230 °C
Scaffold B_I_	B	1250 °C
Scaffold B_II_	B	1210 °C

**Table 2:** Antibodies.

Antibody	Dilution	Catalog N°	Company
anti-PINP	1:1000 in TBS-T	Abx131414	Abbexa, Cambridge, UK
anti-NTX	1:1000 in TBS-T	PAA639hu01	Cloud-Clone, Katy, TX, USA
HRP (horseradish peroxidase)-conjugated secondary antibody	1:10,000 in TBS-T	sc-2004	Santa Cruz Biotechnology, Heidelberg, Germany

**Table 3:** Powder characterization—Particle size analysis.

Particle Size	Powder A	Powder B
D10 (µm)	1.7	1.7
D50 (µm)	4.9	2.6
D90 (µm)	10.9	4.9

**Table 4:** Scaffold characterization (composition determined by X-ray diffractometry, organic residues, inorganic residues).

Powder Characterization	Powder A	Powder B
Organic residues		
Total organic carbon (mg/L)	<1	<1
Total hydrocarbon (mg/L)	<1	<1
Inorganic residues		
Total heavy metals (mg/kg)	<19.5	<11
Crystallography analysis		
Crystallinity ratio (%)	91.34 ± 0.07	99.60 ± 0.37
Foreign Phases *		
Tricalcium phosphate α (%)	1.1 ± 0.2	1.1 ± 0.1
Tricalcium phosphate β (%)	<detection limit	<detection limit
Tetracalcium phosphate (%)	<detection limit	<detection limit
Calcium oxide (%)	<detection limit	<detection limit

* In relation to the crystalline hydroxyapatite phase.

## Data Availability

The original contributions presented in the study are included in the article, further inquiries can be directed to the corresponding author.
